# Fabrication of PES Modified by TiO_2_/Na_2_Ti_3_O_7_ Nanocomposite Mixed-Matrix Woven Membrane for Enhanced Performance of Forward Osmosis: Influence of Membrane Orientation and Feed Solutions

**DOI:** 10.3390/membranes13070654

**Published:** 2023-07-08

**Authors:** Ghadah M. Al-Senani, Mervat Nasr, Mohamed Zayed, Sahar S. Ali, Hind Alshaikh, Hanafy M. Abd El-Salam, Mohamed Shaban

**Affiliations:** 1 Department of Chemistry, College of Science, Princess Nourah bint Abdulrahman University, P.O. Box 84428, Riyadh 11671, Saudi Arabia; gmalsnany@pnu.edu.sa; 2 Nanophotonics and Applications (NPA) Lab, Physics Department, Faculty of Science, Beni-Suef University, Beni-Suef 62514, Egypt; mervat.nasr@science.bsu.edu.eg (M.N.); m.zayed88ph@yahoo.com (M.Z.); 3 Chemistry Department, Faculty of Science, Beni-Suef University, Beni-Suef 62514, Egypt; 4 Chemical Engineering and Pilot-Plant Department, National Research Center, Dokki, Cairo 12622, Egypt; sahar_saad_ali@yahoo.com; 5 Chemistry Department, Science and Arts College, Rabigh Campus, King Abdulaziz University, P.O. Box 344, Jeddah 21911, Saudi Arabia; hfalshakh@kau.edu.sa; 6 Department of Physics, Faculty of Science, Islamic University of Madinah, P.O. Box 170, Madinah 42351, Saudi Arabia

**Keywords:** forward osmosis (FO), membrane (MMW), TiO_2_/Na_2_Ti_3_O_7_ nanocomposite (TNT), PES/(TNT) nanocomposite MMW FO membrane, antifouling membrane, sustainability, volume reduction

## Abstract

Water treatment is regarded as one of the essential elements of sustainability. To lower the cost of treatment, the wastewater volume is reduced via the osmotic process. Here, mixed-matrix woven forward osmosis (MMWFO) PES membranes modified by a TiO_2_/Na_2_Ti_3_O_7_ (TNT) nanocomposite were fabricated for treating water from different sources. Various techniques were used to characterize the TNT nanocomposite. The crystal structure of TNT is a mix of monoclinic Na_2_Ti_3_O_7_ and anorthic TiO_2_ with a preferred orientation of ($\overset{-}{2}11$). The SEM image shows that the surface morphology of the TNT nanocomposite is a forked nano-fur with varying sizes regularly distributed throughout the sample. The impact of TNT wt.% on membrane surface morphologies, functional groups, hydrophilicity, and performance was investigated. Additionally, using distilled water (DW) as the feed solution (FS), the effects of various NaCl concentrations, draw solutions, and membrane orientations on the performance of the mixed-matrix membranes were tested. Different water samples obtained from various sources were treated as the FS using the optimized PES/TNT (0.01 wt.%) MMWFO membrane. Using textile effluent as the FS, the impact of various NaCl DS concentrations on the permeated water volume was investigated. The results show that the MMWFO membrane generated with the TNT nanocomposite at a 0.01 wt.% ratio performed better in FO mode. After 30 min of use with 1 M NaCl and various sources of water as the FS, the optimized MMWFO membrane provided a steady water flow and exhibited antifouling behavior. DW performed better than other water types whenever it was used owing to its greater flow (136 LMH) and volume reduction (52%). Tap water (TW), textile industrial wastewater (TIWW), gray water (GW), and municipal wastewater (MW) showed volume reductions of 41%, 34%, 33%, and 31.9%, respectively. Additionally, when utilizing NaCl as the DS and TIWW as the FS, 1 M NaCl resulted in more permeated water than 0.25 M and 0.5 M, yet a higher volume reduction of 41% was obtained.

## 1. Introduction

Due to climate change and rising industrial and agricultural water demands, the water shortage situation has worsened in many areas of the world. Desalination is sometimes the sole remedy in such deficient locations [1]. Humans must have access to fresh water to survive, so impending water shortages pose a threat to everyone’s quality of life [2]. More efforts have been made to evaluate prospective solutions, such as membrane-based techniques to recover fresh water from seawater and brackish water, in order to tackle this challenge [3]. Currently, reverse osmosis (RO) is a widely used technology for seawater desalination and water filtration. Although RO has dominated for a number of decades, the process faces significant challenges due to fouling issues and high energy consumption [4]. Due to its minimal energy requirements, forward osmosis (FO) is regarded as a modern and environmentally friendly alternative technique. This procedure does not require hydraulic pressure, in contrast to RO. The FO process, a new membrane procedure, uses natural osmotic pressure as the driving force to transfer purified water across the semipermeable membrane between the draw solution, with high osmotic pressure, and the feed solution [5]. Internal concentration polarization (ICP), however, is a severe issue in the FO process that greatly lowers the force that propels water transport [6]. Theoretically, an ideal FO membrane should have a thin support layer with smaller structural characteristics and high porosity to reduce internal concentration polarization, as well as an active layer with high water permeability and low solute permeability [7]. Numerous researchers have recently reported on the valuable impacts of nanoparticle mixed-matrix membranes (MMMs) on the mitigation of membrane ICP and the improvement of the structure. ICP can be minimized by controlling the structural characteristics of the membrane support layer, such as porosity, pore size distribution, tortuosity, and thickness [8,9]. The structural parameter (S value) is typically utilized to indirectly assess the magnitude of the ICP effect in the FO process as an indicative metric of the membrane function [10]. The pumping of fluids to the membrane element is the only energy-intensive part of this osmotic separation, which naturally takes place as a result of the tendency toward thermodynamic equilibrium. It has a significant and well-known influence on the environment due to its minimum energy and material usage [11]. Due to their high susceptibility to fouling, poor performance, and low hydrophilicity, FO membranes have limited industrial uses [12]. Because of this, the majority of FO membranes are typically made using the interfacial polymerization (IP) method to construct thin-film composite (TFC) structures with a specific layer on the support’s top surface [13,14]. This method is used to obtain high-water-flux FO membranes without losing selectivity during the forward osmosis process. In recent investigations, FO membranes with substrates that are thin, low in S value, extremely porous with macrovoids, and very wettable have been developed to reduce ICP and increase water permeability [15,16]. The majority of attempts to create MMW FO membranes have focused on creating a very porous membrane substrate that may have macrovoids resembling fingers and that can be produced by employing lower polymer concentrations during the phase inversion process. Due to their simplicity, flexibility, toughness, and affordable manufacture, polymeric membranes are frequently employed. Nevertheless, they have weak antifouling properties and only moderate mechanical, chemical, and heat resistance [17,18]. Their hydrophobic nature was blamed for their low antifouling capacity. Numerous membrane modification approaches, including bulk modification, surface coating, and mixing, have been developed to improve membrane properties. To enhance the strength, selectivity, permeability, and antifouling properties of polymeric membranes, it may be possible to incorporate an inorganic nanomaterial such as zeolite [14], titanium oxide [19], or carbon nanotubes (CNTs) [20]. Additionally, it was reported that the addition of TNPs to the PES membrane improved the porosity, wettability, chemical stability, and antifouling properties of the formed TNOs/PES FO membranes [21,22]. Previous studies suggest that the presence of numerous hydrophilic functional groups on the surface of TNPs boosts the membrane’s pore width, hydrophilicity, and porosity, which significantly raises the water permeability. TiO_2_ nanoparticles have good chemical stability, photocatalytic activity, and antifouling properties while being inexpensive and non-toxic [23].

Titanium nanotubes have lately attracted interest as potential nanofillers that could enhance the key characteristics of polymeric membranes, namely, their transport, separation, and antifouling capabilities. Titanium nanotubes are highly hydrophilic nanomaterials that exhibit a well-developed porous structure and a large specific surface area [24]. However, there are presently relatively few articles discussing the use of TNTs for producing polymeric membranes. Mahdi et al. [25] attained nanocomposite titanium nanotubes/PES UF membranes with enhanced organic matter rejection and improved pure water flux (PWF) values. According to Shaban et al. [26], the addition of titanium nanotubes to the matrix of polymers improved the membranes’ water permeability, separation, and antifouling capabilities.

Membranes made of polyether sulfone (PES) are frequently employed in the biomedical and water desalination industries. With a high glass transition temperature, the PES membrane has good thermal, mechanical, and oxidative properties. However, the PES membrane’s hydrophobicity, which serves as a barrier in the separation process, may be viewed as a negative characteristic. The phase inversion method is the most widely used method for membrane preparation because it is straightforward and inexpensive and produces a large amount of output. Additionally, this approach is more practical than others for combining various nanostructures with polymer matrices to enhance performance. Titanium nanotubes added to PES increase membrane porosity and improve membrane performance, according to Shaban et al. [27]. Celik et al. [28] stated that the combination of PES with CNTs improves the fouling resistance of membranes. By mixing these nanocomposite FO membranes with the proper proportions of nanofiller, Wang et al. [29] created nanocomposite membranes that performed better than commercial membranes. This was primarily caused by the selective layer’s smoother surface and considerably more porous structure, which led to lower ICP and increased osmotic water flux.

The cost of the treatment process depends on the volume of wastewater to be treated, its application, and its discharge. In this study, we focused on the volume reduction of wastewater using a highly efficient FO membrane prepared under optimal conditions at a low cost. Here, we describe our attempt to prepare ideal membranes for FO application by altering the structure of the CTA membrane by including the TNT nanocomposite with various wt.% ratios in the casting solution. The effect of the TNT nanocomposite on the structure, hydrophilicity, tensile strength, and water flux of these membranes was studied. Our work focused on the modification of the PES membrane for forward osmosis applications by blending it with TiO_2_/Na_2_Ti_3_O_7_ (as a sodium type of ion-exchange resin) to introduce an extra charged passageway. Consequently, water flux, salt rejection, and minimum reverse salt flux will improve. The influence of TiO_2_/Na_2_Ti_3_O_7_ on the PES FO membrane performance was tested using a bench-scale FO setup. The fabricated MMW FO membranes were then employed for volume reduction and membrane fouling with various types of wastewater used as the feed solution and NaCl (1 M) as the draw solution under ideal conditions. Additionally, utilizing textile industry effluent as the feed solution, the impact of various DS concentrations was studied.

## 2. Experimental Information

### 2.1. Raw Materials

Polyether sulfone (PES Ultrason E6020P) was provided by BASF Company, Germany. N, N-Dimethylformamide (DMF), Polyethylene glycol, N-methyl Pyridole (NMP), and TiO_2_ powder were obtained from Lobe Chemie, India. Diluted HCl was supplied by Scharlau, Germany. NaOH was delivered from ADWIC, Egypt.

### 2.2. Woven Support Permeability

Because of an increase in membrane permeability, using woven material (as a support) with a smooth surface and uniform fiber spreading may result in a high permeate flux (i.e., increased open fabric area). Any open mesh fabric can normally support a membrane [30]. During casting, the membrane substance completely envelops the support fabric. The mechanical properties of the woven support are examined and shown in Table S1 (Supplementary Data). The elongation (44.22 mm) and tensile strength (31.21 N/cm^2^) of the woven support were evaluated using a mechanical system (model H5KS Tinius Olsen, Horsham, PA, USA). The measured contact angle was 41 degrees. The thickness and the diameter of the woven cloth were 115 and 11.6 μm, respectively.

The permeability experiment was performed on the fabric support using distilled water (DW) and a filtration lab system with a 5 cm diameter. A feeding hole, a rejection hole, and a permeate hole are all present in this apparatus. The rejection side of the membrane faced the applied pressure. The permeability of the woven cloth was nearly 1254 (m^2^h.bar).

### 2.3. Manufacture and Characterization of TiO_2_/Na_2_Ti_3_O_7_ (TNT) Nanocomposite

The TNT nanocomposite was fabricated using a straightforward alkaline hydrothermal method followed by a calcination process. A total of 4 gm of TiO_2_ (powder) was suspended in 400 mL of 10 M NaOH and stirred vigorously for 40 min. Then, the subsequent solution was poured into a 1 L Teflon-lined stainless-steel autoclave for hydrothermal management at 160 °C for 1 day in a muffle furnace. After the complete hydrothermal reaction, the autoclave cooled at room temperature, and the obtained white precipitate was separated. After that, the product was filtered and washed with 0.1 M HCl and distilled water (DW) several times to remove the unreacted Na^+^ ions. Finally, the produced white powder was dried at 80 °C for 4 h and calcined at 450 °C for 2 h. The prepared TNT nanocomposite was characterized using various analytical techniques. The X-ray diffraction technique (XRD; PANalytical X’Pert Pro, Westborough, MA, USA) was used to investigate the structural properties of the prepared TNT nanocomposite. Morphological analyses were examined by using a scanning electron microscope (SEM; FEI Quanta 200, Eindhoven, Holland) and a scanning transmission electron microscope (STEM; ZEISS SUPRA 55 VP, Troy NY, USA). The purity of the TNT nanocomposite powder was checked by using a Raman spectrometer (Thermo Fisher Scientific, model DXR3xi, Gemini, Altrincham, UK) with a 532 nm laser.

### 2.4. Preparation of PES/TNT Nanocomposite MMW FO Membranes

PES/TNT nanocomposite MMW membranes were prepared by the phase inversion process and are now prepared for use in a variety of FO applications [31]. Different chemical ratios (22 wt.% PES, 0.5 wt.% PEG) and the TNT nanocomposite with different wt.% ratios were dissolved in NMP solvent with various weight ratios, as mentioned in Table 1. The following procedures were used: To achieve a consistent dispersion of the TNT nanocomposite in the casting solution, different wt.% of the TNT nanocomposite and 0.5 wt.% PEG were first dispersed in various wt.% of NMP solvent by utilizing ultrasonic bath equipment for 4 h at room temperature. Second, to achieve homogeneity, 22 wt.% PES was added to the produced casting solution and stirred mechanically for 2 h at room temperature. The casting solution was then left to stand for 24 h to eliminate any bubbles and prepare it for casting to obtain the membrane. Using a casting knife, the casting solution was finally cast on a woven fabric (support material) with a casting thickness of 215 μm, and then it was immediately submerged in water as a non-solvent solution. The manufactured membranes were kept in a brand-new distilled water bath for 1 day until the phase inversion process was guaranteed to be complete, at which point the membrane was disengaged from the glass bowl. To remove any remaining solvent, the membrane was peeled away and then washed with DW. After being maintained in fresh DW, the generated membrane was then used for further characterization.

### 2.5. Characterization of PES/TNT Nanocomposite MMW FO Membrane

#### 2.5.1. Morphology and Functional Groups

Field-emission scanning electron microscopy (FESEM, ZEISS SUPRA 55 VP, Gemini, Altrincham, UK) was used to capture pictures of membrane samples under a scanning electron microscope (SEM). Before testing, membrane samples were dried in an oven set to 30 °C. For cross-section SEM imaging, a single precise blow was used to cut the membrane. Surface roughness was analyzed with the ImageJ program (v5.0.3, National Institutes of Health, Rockville, MD, USA). The functional groups of the fabricated membranes were identified using Fourier transform infrared spectroscopy (ATR-FTIR, VERTEX 70, Bruker, Billerica, MA, USA) in the range of 600–4000 cm^−1^. 

#### 2.5.2. Water Uptake Rate, Contact Angle, Tortuosity, Porosity, and Structural Parameter

The contact angle was measured using the sessile drop technique via a CAM 300 Optical Contact Angle Meter (KSV Instruments Inc., Connecticut, CT, USA) system. A microsyringe was used to place a 5 μL droplet of deionized (DI) water on the membrane’s surface. To gauge the angle that developed at the liquid–solid contact, the droplet’s photograph was taken. For each sample, to reduce error, the contact angle was measured four times, and the average value was computed. In defining a membrane’s hydrophobicity and membrane fouling, the contact angle is crucial. For PES and PES/TNT nanocomposite MMW FO membranes, the physical characteristics, including membrane water uptake, contact angle, tortuosity, porosity, and structural parameter, were estimated.

Modified PES/TNT nanocomposite MMW FO membranes’ water uptake rate and porosity were assessed using the weight change following hydration. The membrane was first pre-soaked in deionized water at room temperature for 24 h. The membrane was quickly weighed to determine the weight of the wet membrane after the surface had been cleaned with filter paper (W_W_). The wet membranes were then dried for 24 h at 80 degrees Celsius in an air-circulating oven before being weighed (W_d_). The water uptake percentage was determined from the following formula [32]:

(1) $$Water\;uptake\left(\%\right)=\frac{\left(W_{w}-W_{d}\right)}{W_{d}}\times 100$$

The porosity (P) of fabricated PES/TNT nanocomposite membranes was evaluated by using the following formula [33]:

(2) $$Porosity(\%)=\frac{\left(W_{w}-W_{d}\right)}{V}\times 100$$

where W_w_ is the weight of wet membranes (g), and W_d_ is the weight of dry membranes (g). The volume can be determined using the equation V = At, where A (cm) and t (cm^2^) are the thickness and the surface area of the synthesized membrane, respectively.

Membrane tortuosity (τ) can estimate by using the porosity value from the following equation [34]:

(3) $$\tau =\frac{{\left(2-\varepsilon \right)}^{2}}{\varepsilon }$$

The structural parameter (S) of the membrane is a critical property of any osmotic membrane. The S parameter depends on the tortuosity (τ), the thickness of the support layer (t), and its porosity (*ε*) according to the following relation [35]:

(4) $$S=\frac{t.\tau }{\varepsilon }$$

### 2.6. The Performance of PES/TNT Nanocomposite MMW FO Membrane

The performance of the produced PES/TNT nanocomposite MMW FO membranes was examined using a bench-scale FO system module. The effective filtration area was 56 cm^2^ for the examination of the performance of a membrane in FO mode. DW acts as a feed (FS) solution facing the active layer (polished surface of membrane), and NaCl (1 M) acts as a draw solution (DS) facing the other layer. All FO analysis experiments were performed at room temperature using a constant crossflow rate value (0.857 L/min). The following equations were used to estimate the FO membrane performance in terms of water flux (J_w_), reverse salt flux (RSF) (J_s_), specific reverse salt flux (SRSF) (J_s_/J_w_), and salt rejection (SR):

(5) $$\mathrm{The}\;\mathrm{water}\;\mathrm{flux}\;(J_{w})=\frac{\Delta V}{A\times \Delta t},\;(L/m^{2}\;h)$$

Here, ΔV is the volume of water moved via the FO process in liters (L) during a time interval of Δt in hours (h), and A is the effective surface area of the membrane in m^2^.

(6) $$\mathrm{The}\;\mathrm{reverse}\;\mathrm{salt}\;\mathrm{flux}\;(J_{s})=\frac{C_{t}V_{t}-C_{0}V_{0}}{A\times t},\;(g/m^{2}\;h)$$

where C_o_ and C_t_ are the feed solution’s initial and ultimate salt concentrations, respectively. Additionally, V_t_ and V_0_ (L) stand for the feed solution’s (FS) final and beginning volumes, respectively.

(7) $$\mathrm{Specific}\;\mathrm{reverse}\;\mathrm{salt}\;\mathrm{flux}\;(SRSF)=\frac{J_{S}}{J_{w}},\;(g/L)$$

(8) $$\mathrm{Salt}\;\mathrm{rejection}\;(SR)=\left(1-\frac{C_{p}}{C_{f}}\right)\times 100,\;(\%)$$

where C_f_ represents the concentration of the feed solution, and C_p_ symbolizes the concentration of the permeate solution. The average values are reported with standard deviation values, and all measurements were made in triplicate at room temperature. (Error bars correspond to the standard variance of measurements made in triplicate.)

#### 2.6.1. Effect of TNT wt.% Ratio on the PES MMWFO Membrane Performance

The performance of the PES/TNT nanocomposite MMW FO membrane was examined in the FO system in the FO mode, with the shiny phase facing the feed solution (DW) and the other side facing 1 M NaCl. The effect of different wt.% ratios of the TNT nanocomposite (0 to 0.1 wt.%) on the performance of the PES/TNT MMW FO membrane fabricated with 22 wt.% PES and 0.5 wt.% PEG as pore-forming materials with a casting height of 215 µm was examined.

#### 2.6.2. Effect of Loaded TNT wt.% Ratios and PES/TNT MMWFO Membrane Orientations on FO Performance

With 1 M NaCl used as the draw solution (DS) and distilled water used as the feed solution (FS), the effects of the loaded TNT (0 to 0.1 wt.%) @ PES membrane and the MMWFO membrane orientations (PRO and FO mode) on the FO membrane performance (J_w_ LMH, J_s_ GMH, and SR%) were investigated. The PRO mode (shiny side of the membrane facing the DS, AL-DS) and FO mode (shiny side of the membrane facing the FS, AL-FS) of each manufactured FO membrane were both tested.

#### 2.6.3. Impact of Different NaCl Concentrations as DS on the Performance of Optimal ST3 Membrane

The effect of various NaCl concentrations (0.6, 1, 1.5, 2, and 2.5 M) as the DS and distilled water as the FS on the performance of the FO-modified membrane was tested. FO mode was used for the procedure.

### 2.7. MMWFO Membrane (ST3) Applications

#### 2.7.1. Effect of Various Feed Types on the Performance of MMWFO Membranes

The impact of several feed types (distilled water, tap water, textile industrial effluent, and municipal wastewater) on the best-performing MMWFO membrane created under ideal conditions utilizing 1 M NaCl as the DS was investigated. The parameters of the final mixture of low-strength gray water, municipal wastewater, and industrial textile wastewater collected from a factory in Cairo are shown in Table 2 (average values of various samples).

#### 2.7.2. Effect of Time on the Flux of ST3 Membrane

Distilled water and various types of wastewater were used as the FS, whereas 1 M NaCl was used as the DS to study the impact of time (up to 3 h) on ST3 FO membrane flux.

#### 2.7.3. Effect of Different NaCl Concentrations on the Volume Reduction of Textile Wastewater

The volume reduction of industrial wastewater from the textile industry and water reuse for the dilution of various concentrations in response to various NaCl concentrations (0.25, 0.6, and 1) M as the DS were studied.

## 3. Results and Discussion

### 3.1. Characterization of Fabricated TNT Nanocomposite

**Figure 1:**
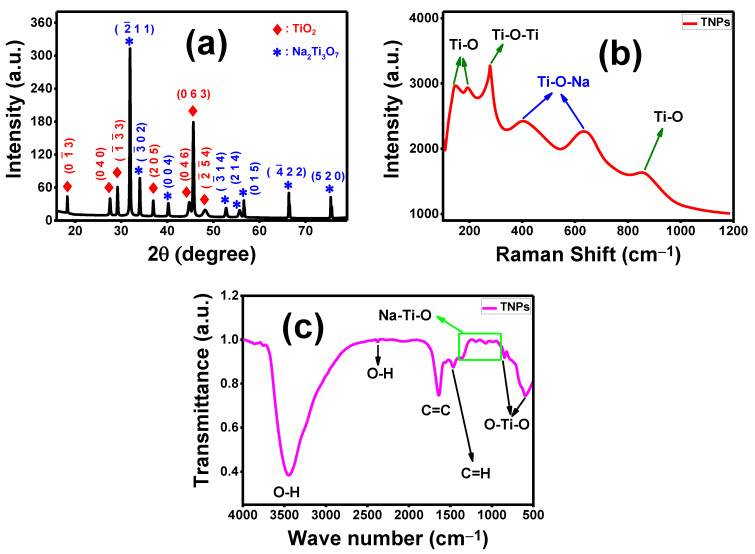
TNT nanocomposite characterization: (**a**) XRD chart, (**b**) Raman spectrum, and (**c**) FTIR analysis.

The XRD chart, Raman spectrum, and FTIR spectrum of the hydrothermally fabricated TNT nanocomposite are illustrated in Figure 1.

#### 3.1.1. Structural Analysis of TNT Nanocomposite

XRD was used to study the crystallographic structural parameters of the fabricated TiO_2_ nanostructure. Figure 1a illustrates the XRD pattern of the TNT nanocomposite. It is clear that the TNT nanocomposite has good crystallinity, and there are characteristic peaks for monoclinic Na_2_Ti_3_O_7_ and anorthic TiO_2_, as indicated in Figure 1a. The characteristic peaks confirming the presence of TiO_2_ with an anorthic lattice are positioned at 2θ = 18.33°, 27.57°, 29.20°, 36.95°, 44.75°, 45.63°, and 48.17°, associated with the Miller index planes ($0\overset{-}{1}3$), (040), ($\overset{-}{1}\overset{-}{3}3$), (205), (046), (063), and ($\overset{-}{2}\overset{-}{5}4$), respectively, according to card no. 96-412-4499 [36]. For monoclinic layered sodium titanate (Na_2_Ti_3_O_7_), there are several distinguishing peaks detected at diffraction angles of 2θ = 31.91°, 34.03°, 40.21°, 52.73°, 55.67°, 56.62°, 66.37°, and 75.47°, attributed to planes ($\overset{-}{2}11$), ($\overset{-}{3}02$), (004), ($\overset{-}{3}14$), (214), (015), ($\overset{-}{4}22$), and (520), respectively, according to card no. 96-231-0332 [37].

Several important XRD parameters, namely, the d-spacing (*d*), the crystallite size (*D*), the dislocation density (δ), and the texture coefficient (TC), were calculated and are listed in Table 3. The inter-planar distance (d-spacing) can be estimated from the angle of diffraction according to Bragg’s equation [38]:

(9) $$d=\frac{n\lambda }{2sin\theta }$$

where n, λ, θ, and *d* are the constant diffraction order (n = 1, 2, ….), the incident X-ray wavelength, the diffraction angle, and the d-spacing, respectively.

The average crystallite size (*D*) of the TNT nanocomposite was assessed, as listed in Table 3, by using Scherrer’s equation [39]:

(10) $$D=\frac{0.94\lambda }{\beta cos\theta }$$

where β is the full half-width maximum obtained from XRD data. The average crystallite size values are nearly 52.6 and 70.5 nm for TiO_2_ and Na_2_Ti_3_O_7_, respectively, as listed in Table 3. The conventional explanation for the smaller crystallite size is the expansion of more nucleating centers as the nucleation energy barrier is lowered [24].

The dislocation density (δ) of the fabricated TiO_2_ nanostructure, which describes the number of defects in the sample, is a well-defined measurement of dislocations in a crystalline sample. Since a dislocation is a link defect, this is defined as the overall length of the dislocation rows per unit volume (L/L^3^) of the sample. The dislocation density (δ) was determined by using the following relation [40]:

(11) $$\delta =\frac{1}{D^{2}}$$

As shown in Table 3, the dislocation density is very low (0.3 × 10^−3^ dis/nm^2^), which indicates few defects in the fabricated TNT nanocomposite.

The texture coefficient (TC) denotes the texture of a particular plane. TC > 1 indicates the preferred direction of growth. The TC for the TNT nanocomposite planes is calculated from the following relation [41]:

(12) $$TC\left(hkl\right)=\frac{I\left(hkl\right)/I_{0}\left(hkl\right)}{\left(N^{-1}\right)\left[\sum \frac{I\left(hkl\right)}{I_{0}\left(hkl\right)}\right]}$$

where I(hkl) is the measured intensity, I_o_(hkl) is the standard intensity value obtained from the JCPDS card for a plane (hkl), and N is the number of observed diffraction peaks. For TiO_2_, the (063) peak has the greatest intensity, and its TC value is 3.58, while for Na_2_Ti_3_O_7_, the ($\overset{-}{2}11$) peak is the most intense, with a TC of 4.31. The highest TC values suggest that anorthic TiO_2_ and monoclinic Na_2_Ti_3_O_7_ grow perfectly along the (063) and ($\overset{-}{2}11$) orientations, respectively. Finally, the average crystallite size for the TiO_2_/Na_2_Ti_3_O_7_ nanocomposite is very small (~60 nm), which suggests a high surface area for reactions and better performance in the FO application.

#### 3.1.2. Raman Spectroscopy

To confirm the composition of the TNT nanocomposite, along with XRD data, the Raman spectrum was measured in the 50–1000 cm^−1^ range using the Raman spectroscopy technique. Figure 1b shows the Raman spectrum of the fabricated TNT nanocomposite. The Raman spectrum of the TNT nanocomposite exhibits a typical basic peak related to Ti-O-Ti stretching vibration at 278 cm^−1^, and three characteristic peaks attributed to Ti-O stretching vibrations located at 142, 190, and 860 cm^−1^. There are also two peaks associated with Ti-O-Na vibration, positioned at 397 and 620 cm^−1^. Our data on the TNT nanocomposite spectrum agree with previously reported research on TiO_2_ Raman spectra [42,43]. These results indicate the strong binding between TiO_2_ and Na_2_Ti_3_O_7_, which confirms the XRD data.

#### 3.1.3. Infrared Spectroscopy (FTIR) Analysis of TNT Nanocomposite

The FTIR technique was used to identify the chemical structure and the functional groups of the TiO_2_/Na_2_Ti_3_O_7_ nanocomposite via computerized spectroscopy in the range 500–4000 cm^−1^. Figure 1c shows the FTIR spectrum of the TNT nanocomposite fabricated using the hydrothermal method. The FTIR spectrum (Figure 1c) revealed peaks related to TiO_2_ and Na_2_Ti_3_O_7_. The peak observed at 590 cm^−1^ is due to the vibration of the O-Ti-O bond [44], which is the characteristic peak of TiO_2_. In addition, the Ti=O asymmetric stretching peak occurs between 800 and 900 cm^−1^, typical of oxygen-bridged titanium (IV) oxides [45]. There are other functional groups located at 2370 cm^−1^ and 3440 cm^−1^ assigned to the O-H group and O-H stretching, respectively, which confirm the hydrophilic nature of the TNT nanocomposite, as shown in Figure 1c. These obtained results agree with the data supplied by G. A. Yahya et al. [46], H. A. Silim et al. [47], G. P. Singh et al. [48], K. M. S. Khalil [49], and V. Sharma et al. [50] indicating that the bands at 2370 and 3440 cm^−1^ are due to the stretching vibration of oxygen-containing hydroxyl (O-H) functional groups adsorbed from water molecules. The minor bands ranging from 900 to 1300 cm^−1^ may be ascribed to different kinds of Na-titanate mixtures [45,51]. Other features could also be assigned to symmetric C=H stretching, observed at 1460 cm^−1^ [52,53,54]. The FTIR results are in agreement with XRD and Raman patterns for the TNT nanocomposite, which is a combination of TiO_2_ and Na_2_Ti_3_O_7_ nanoparticles. Finally, the fabricated TNT nanocomposite has good hydrophilic properties due to the existence of hydroxyl and carbonyl and, when mixed with the PES matrix membrane, will enhance permeability and antifouling properties for different FO applications.

#### 3.1.4. SEM and TEM Analysis for Fabricated TNT Nanocomposite

**Figure 2:**
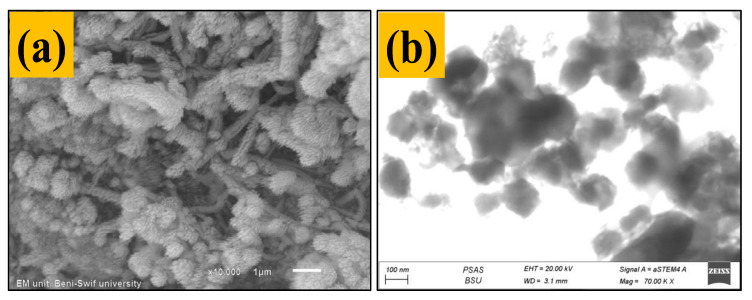
Morphological study of TNT nanocomposite: (**a**) SEM image and (**b**) TEM image.

The morphology of the as-synthesized TNT nanocomposite was studied by SEM and TEM techniques, and the obtained results are presented in Figure 2. The SEM image shows that the surface morphology of the TNT nanocomposite is a forked nano-fur, similar to hair-covered plant and insect surfaces, with varying sizes regularly distributed throughout the TNT sample, as shown in Figure 2a. In addition, it is observed that some of the surfaces have not yet been converted to fur and appear as nanoribbons. It is known that the nano-fur morphology is superhydrophobic and has a high surface area [55], which is good for FO membrane applications. The TEM analysis results, shown in Figure 2b, show that the TNT nanocomposite surface is an agglomeration of nanoparticles distributed throughout the sample, which means that it was not straightforward to find single monolayers. The agglomerated particles appear dark and spherically shaped, like spongy dots.

### 3.2. PES/TNT Nanocomposite MMWFO Membrane Characterization

#### 3.2.1. FTIR and Functional Group Analysis of PES/TNT Nanocomposite MMWFO Membranes

**Figure 3:**
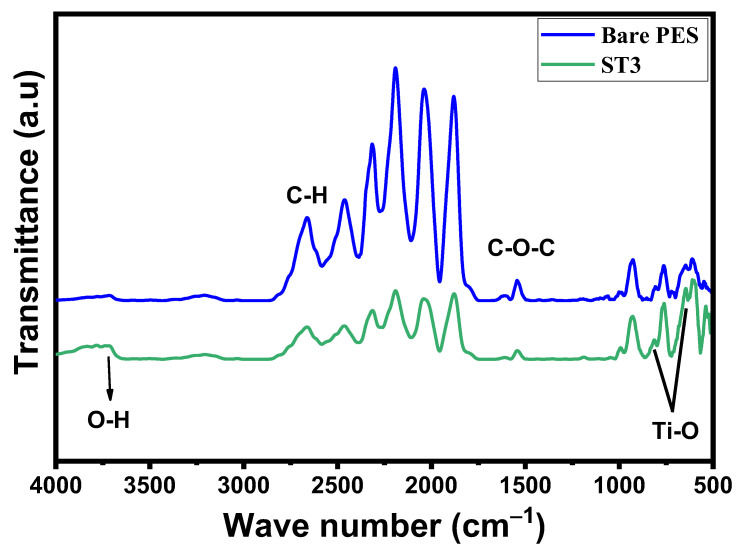
FTIR spectrum of bare PES membrane and optimal PES/TNT nanocomposite MMW FO membrane with loaded TNT nanocomposite at a 0.01 wt.% ratio.

An FTIR investigation was performed to assess the differences in the functional groups of fabricated PES/TNT nanocomposite membranes due to the incorporation of the TNT nanocomposite into the polymeric matrix. Figure 3 shows the FTIR spectra for manufactured bare PES membranes and MMW membranes with the incorporation of 0.01 wt.% TNT nanocomposite. The chemical construction of polyether sulfone (PES) includes three important functional groups containing benzene, ether, and sulfone [56]. There are several peaks detected in the IR spectrum linked to PES. The peak positioned at 1295 cm^−1^ arises due to sulfonic group (O=S=O) stretching vibration, while the symmetric stretching of O-S-O gives a band at nearly 1150 cm^−1^. In addition, the presence of two characteristic peaks at around 1040 cm^−1^ and 1240 cm^−1^ are consistent with symmetric and asymmetric stretching vibrations of the C-O-C group, respectively. The bands between 2900 cm^−1^ and 3100 cm^−1^ represent aliphatic and aromatic C-H stretching vibrations [57]. There are other minor peaks related to benzene and ether groups, which are the main components of PES. For the PES/TNT nanocomposite (0.01 wt.%), intense Ti–O bands were found at wave numbers ranging from 500 to 800 cm^−1^, which is the characteristic peak of TiO_2_. Due to the presence of the O–H functional groups in the TNT nanocomposite, the hydrophilicity of the PES/TNT nanocomposite membrane surface is believed to be enhanced, and the membrane features for FO application efficiency will increase.

#### 3.2.2. Surface Morphology of PES/TNT MMWFO Membranes

**Figure 4:**
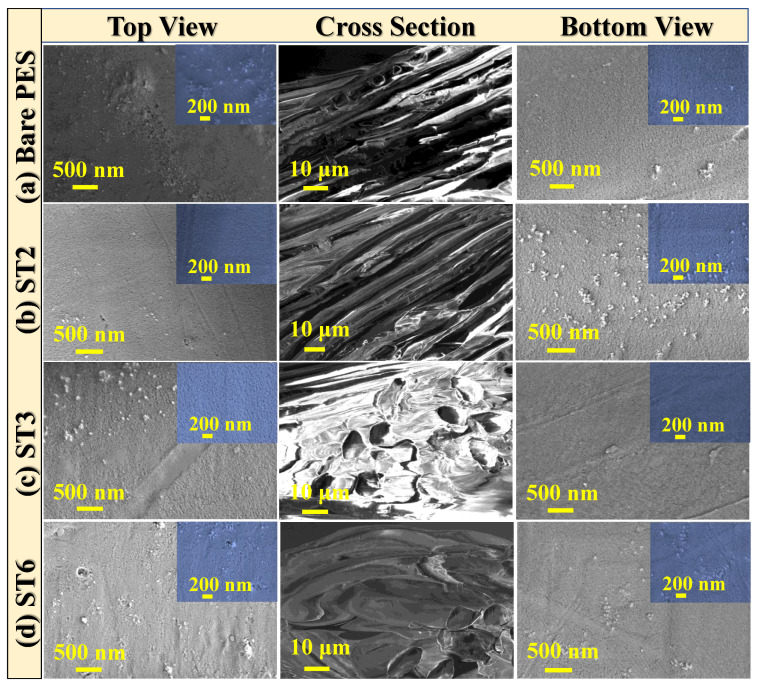
Morphological study: Top, cross-sectional, and bottom views of PES/TNT MMWFO membranes with different TNT nanocomposite wt.% ratios: (**a**) 0 wt.%, (**b**) 0.008 wt.%, (**c**) 0.01 wt.%, and (**d**) 0.1 wt.%.

Scanning electron micrographs (Figure 4) helped to deepen and clarify our understanding of how well the re-growth procedure reorganized the loaded TNT nanocomposite on the top, bottom, and cross-sectional surfaces of PES MMW membranes. As shown in Figure 4a–d, there are considerable differences between the surfaces of the pristine PES and PES/TNT nanocomposite MMW membranes. The images indicate that TNT nanocomposites are incorporated into the top and bottom surfaces of the PES membranes. This might be a result of the hydrophilic nature of the TNT nanocomposite, which is crucial for the best solvent and non-solvent exchange during the manufacturing process [58]. This, in turn, tends to create nanopores in the membrane surface and leads to changes in the cross-sectional surface, as shown in ST3 macrovoid structure [59,60].

From the top-surface SEM views, all membranes contain dense top surfaces with limited nanoporous features. These nanopores became enlarged with increasing wt.% ratios of the loaded TNT nanocomposite from 0 to 0.1 wt.%, as displayed in the inset magnified images (Figure 4). In addition, the pore size decreased with increasing wt.% ratios of the TNT nanocomposite loaded into PES, confirming that the TNT nanocomposite was well dispersed in the polymer matrix. Additionally, as shown in Figure 4d, we noticed the existence of TNT nanocomposite agglomeration on the membrane surface at higher TNT nanocomposite wt.% ratios.

The cross-sectional images of the fabricated PES/TNT nanocomposite MMW membranes revealed that the morphology was critically influenced by the addition of the TNT nanocomposite, as shown in Figure 4. The cross-section image of pristine PES (Figure 4a) shows a unique dense structure, which is also influenced by the loading of the TNT nanocomposite onto the PES membrane during the phase separation method (Figure 4b). Numerous pores embedded in the woven fiber expanded from the top surface to the bottom with increasing TNT nanocomposite wt.% ratios up to 0.01 wt.% (ST3) (Figure 4c). This could be due to the creation of a peel layer that was induced by the phase inversion procedure. The presence of nodules on the top and bottom surfaces of PES/TNT nanocomposite membranes could indicate the successful embedment of nanofillers throughout the membrane structure [57], which, in turn, improves the water permeability features. When loaded at a high wt.% ratio (0.1 wt.% at ST6), the TNT nanocomposite was agglomerated on the membrane surface and coated most of the woven fibers, as manifested in the cross-sectional structure in Figure 4d.

Generally, the porous layer structure of all produced membranes was uneven, with a dense top layer, and the porous layer structure extended from the middle to the bottom surface. As indicated in the insets of Figure 4a–d, the pore size of PES/TNT nanocomposite membranes was reduced compared to that of the bare PES membrane with increasing wt.% of the loaded TNT nanocomposite up to ST3 (0.01 wt.%), and thereafter, it decreased at higher wt.% of the loaded TNT nanocomposite. As a result, the surface area and hydrophilic properties of the optimal ST3 membrane were improved.

#### 3.2.3. Atomic Force Microscopy (AFM) Analysis and Roughness Parameters

**Figure 5:**
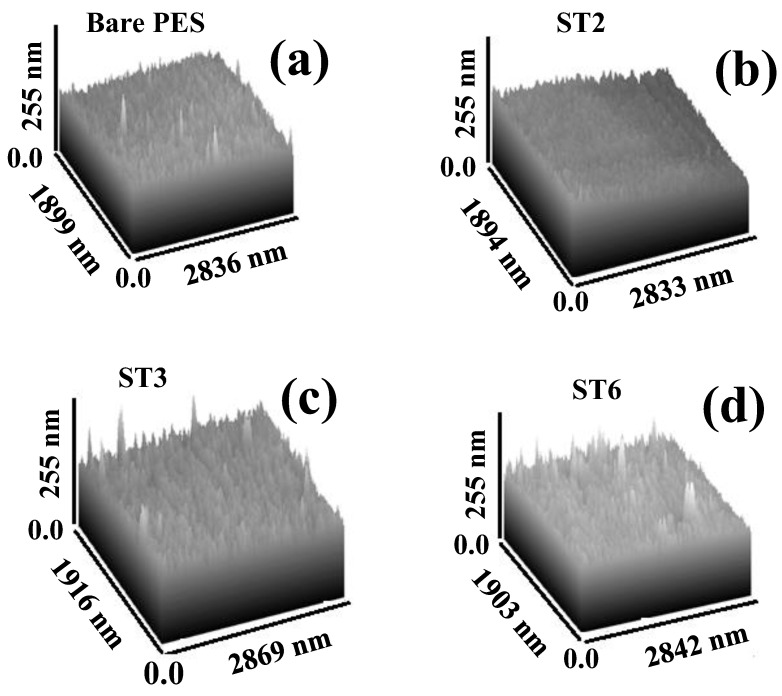
Three-dimensional AFM images of the fabricated PES/TNP MMW FO membranes with different TNP wt.% ratios: (**a**) 0 wt.%, (**b**) 0.008 wt.%, (**c**) 0.01 wt.%, and (**d**) 0.1 wt.%.

An AFM device was used to characterize the three-dimensional morphology and surface roughness of the fabricated bare PES and PES/TNP MMW membrane, as illustrated in Figure 5. The estimated surface roughness parameters of the top and bottom for the prepared membrane are presented in Table 4. For roughness, the top and bottom surface roughness of fabricated PES/TNP MMW membranes increased when increasing the wt.% of the loaded TNT from 0 to 0.01 wt.%, as shown in Figure 5 and listed in Table 4. These results may be due to the hydrophilic nature of TNPs, which cause a fast phase exchange during the phase inversion manufacturing process, allowing the nanopores to disperse throughout the membrane from the top to the bottom surfaces, as indicated in the SEM images (Figure 4). The obtained AFM results confirm that the TNPs were completely incorporated into the polymer membrane matrix, which is consistent with the SEM image analysis.

In Table 4, the Ra and Rq parameters indicate the average roughness and the average square root roughness of the scanning top and bottom surfaces. According to Table 4, overall, the roughness of the fabricated PES/TNP membranes (Ra and Rq) was enhanced due to the addition of TNPs to the polymer matrix, which, in turn, increased the surface area for water transport, the pore size, mechanical strength, and stability. The surface roughness of the membrane is a very critical parameter in the enhancement of the antifouling properties of the membrane. In addition, the addition of TNPs could enhance the viscosity of the polymeric casting solution. Much research has been performed on this phenomenon in which increased viscosity produces additional holes, similar to the pores seen in this study [61,62].

## 4. Evaluating the Role of TNPs in Improving Membrane Contact Angles, Water Uptake, and Porosity Measurements of Membrane

### 4.1. Contact Angles

The water contact angle was used to examine the surface hydrophilicity (wettability) of the fabricated membrane, as shown in Table 5. Table 5 reports the influence of TNP loading on the surface contact angle (hydrophilicity) of the PES/TNP MMW FO membranes fabricated in our research. The results indicate that as the TNT loading was increased from 0 to 0.1 wt.%, the contact angle of the PES substrate tended to drop from 78 to about 51°. The hydrophilicity of the embedded TNPs is what causes the surface contact angle to be significantly reduced. Pristine PES has the greatest contact angle of 78° due to its hydrophobic nature, while the sample containing 0.1 wt.% showed the lowest contact angle of 51° for NMP. These findings confirm that the related PES/TNT MMW membranes have improved surface hydrophilicity in comparison to the pure PES membrane [63]. Due to the addition of hydrophilic nanoparticles, the surface hydrophilicity and surface morphology (dense pores) of PES/TNT MMW FO membranes increased, consistent with the results that have been reported in the literature [64]. As was already noted, the migration of the hydrophilic TNPs to the membrane surfaces during membrane formation processes (phase inversion) is responsible for this enhancement [65].

### 4.2. Water Uptake

The water uptake measurement is one more definitive study to comprehend the hydrophilic character of the membrane and is shown in Table 5 for the manufactured PES/TNT nanocomposite MMW membrane. First, the quantity of hydrophilic sites in the membrane matrix affects how much water the membrane can absorb. Second, water uptake is affected by the membrane shape and the fact that the polymeric membrane sub-layer contains dense pores that are embedded in woven fibers. This is evident from the water uptake results shown in Table 5. Equation (1) was used to determine water uptake. The water uptake of the pure membrane was 49%, while it was 77% after adding TNT in amounts up to 0.01 wt.% for NMP. As the concentration of TNT doping increases in the polymer matrix, the water uptake is enhanced. Therefore, it may be said that as TNT doping increases, the membrane’s attraction for water increases. As shown by FTIR and the surface morphologies of the produced FO membranes, TNTs have hydrophilic spots in them, which enable the uptake of the solvent (water) into the membrane matrix [66].

### 4.3. Porosity

All manufactured PES/TNT nanocomposite MMW FO membranes had significant porosity, ranging from 54% to 74% for TNTs with wt.% up to 0.01% for NMP, compared to 45% for the pure PES membrane with NM. These findings show that the porosity of PES/TNT nanocomposite MMW FO membranes increases as TNT wt.% ratios increase [67]. This means that, similar to TNTs, the porosity of the membrane’s internal structure improves, improving the membrane’s characteristics and potentially contributing to an increase in the membrane’s directional flow rates [68]. Additionally, the ST3 membrane exhibits the lowest tortuosity and S value. 

## 5. Membrane Performance

### 5.1. Effect of Loaded TNT wt.% Ratios and PES/TNT MMWFO Membrane Orientations on FO Performance

**Figure 6:**
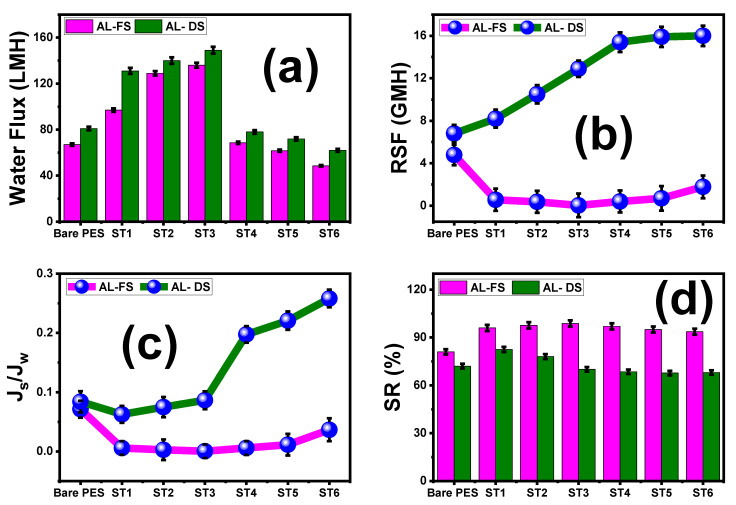
FO membrane performance. Comparison of (**a**) water flux (J_w_), (**b**) reverse salt flux (J_s_), (**c**) specific reverse salt flux (J_s_/J_w_), and (**d**) salt rejection (SR%) in both FO and PRO modes of the prepared MMWFO membranes. The standard deviation of measurements taken in triplicate is indicated by error bars.

Figure 6 shows a comparison of J_w_, J_s_, J_s_/J_w,_ and SR between the FO and PRO modes of the fabricated MMWFO membranes with different TNT wt.% ratios using 1 M NaCl solution as the DS and DI water as the FS. The obtained values of water flux (J_w_), reverse salt flux (J_s_), specific reverse salt flux (J_s_/J_w_), and salt rejection (SR%) for all fabricated membranes in both the PRO (AL-DS) mode and FO (AL-FS) mode are listed Table 6. The flux of the membranes increased with increasing TNT ratios until ST3 (0.01 wt.%) and then decreased with any further increase in the TNT wt.% (Figure 6a). For the ST3 membrane, the maximum observed Jw value was 149 LMH in PRO mode and 136 LMH in FO mode. With the addition of TNTs, the trend of rising J_w_ closely matches the rising hydrophilicity [69]. This difference between the PRO and FO modes of the process’s water flux (J_w_) is caused by the way in which the membrane’s structural resistance is applied to the sides of the solutions that it supports [70]. The lack of internal concentration polarization (ICP), which significantly affects water flux in FO mode, as well as the mesh osmotic pressure reduction when using DI water as the feed solution facing the support layer (SL) of the prepared MMWFO membrane, is the reason why the obtained Jw values in PRO mode are higher than those in FO mode [71]. When the draw solution (DS) in PRO mode faced the glossy face of the membrane, dilutive external concentration polarization (d ECP) caused ICP to occur in reverse when the system was operating in PRO mode. Additionally, it is noted that the application of the PRO mode for desalination is not practicable due to harsh fouling because the porous support layer (SL) facing the feed solution contains scaling and fouling particles, despite the PRO process attaining greater J_w_ in bench-scale tests under ideal conditions [72]. The obtained high water fluxes could be attributed mainly to improved surface hydrophilicity and partly to an increased effective surface area (rougher surface, Figure 5). Moreover, the interconnected nanoporous channels extending from the top surface to the bottom (ST3, Figure 4) could further enhance the permeation of water molecules through the membrane [73,74]. In addition, the improved performance values are consistent with the other membrane parameters, such as porosity, contact angle, water uptake, and membrane morphological studies. In addition, Na_2_Ti_3_O_7_ behaves as an ion-exchange resin, which is confirmed by the absence of any pollutants in the DS solution. Similarly high water flux values have been reported in the literature [75,76,77]. According to P. Sukitpaneenit et al. [75], because the membrane support has smaller pores and a narrower pore size distribution, TFC-FO-PES_water/NMP/PEG_ membranes exhibit a high water flux up to 34.5 and 65.1 LMH with a 2.0 M NaCl DS in FO and PRO modes, respectively. N. Widjojo et al. [76] reported that TFC-FO membranes with a more hydrophilic and fully sponge-like structure, i.e., sulfonated polyphenylene sulfone materials, exhibit much higher water flux with a value of 54 LMH in PRO mode using 2 M NaCl as a DS. According to Q. Liu et al. [77], the PES/PAA5/CaCO3 membrane’s highest water flux was 52 LMH and 62 LMH in the AL-FS and AL-DS modes, respectively, because of the significant ICP reduction brought on by the improved water and salt transport. The resulting RSF (J_s_) values in FO(Al-FS) mode were lower than the obtained values in PRO(AL-DS) mode utilizing all developed membranes, as shown in Figure 6b and Table 6. According to the findings, the PRO form of the process produces a stronger driving force than the FO process because the draw solute is more concentrated on the membrane surface. On the other hand, with neat bare PES and prepared MMW-modified membranes, the reason why there is more concentrated DS on the membrane surface in PRO mode also causes RSF to increase. Figure 6b demonstrates that Js in the PRO process is greater than in FO mode, which matches the results obtained by Lambrecht et al. [78]. In addition, the specific reverse salt flux (J_s_/J_w_) in PRO mode is higher than in FO mode, as shown in Figure 6c. As shown in Figure 6d, the salt rejection rises from 81% to 98.8% for AL-FS while rising from 72% to 82.5% for AL-DS, as listed in Table 6.

### 5.2. Effect of Various NaCl Concentrations on the Performance of ST3 Membrane

**Figure 7:**
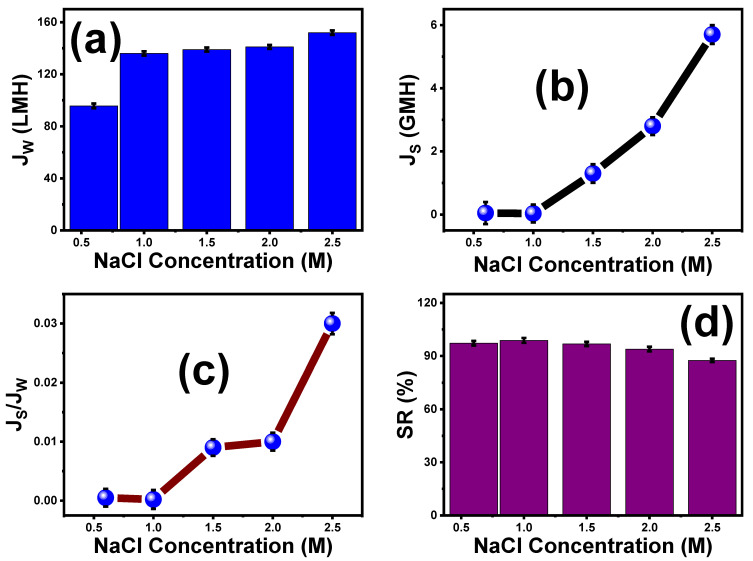
The influence of different NaCl concentrations on ST3 FO membrane performance: (**a**) water flux (J_w_), (**b**) reverse salt flux (J_s_), (**c**) specific reverse salt flux (J_s_/J_w_), and (**d**) salt rejection (SR%). The standard deviation of measurements taken in triplicate is indicated by error bars.

The impact of various NaCl concentrations on the optimal ST3 membrane function is depicted in Figure 7. With increasing NaCl concentrations (0.5 to 2.5 M), the water flux (J_w_) increased until reaching a maximum of 152 LMH at a 2.5 M salt concentration, as shown in Figure 7a. The stronger pushing force across the membrane caused by the concentration difference between the DS and FS caused the FO water flux to increase with increasing draw solution concentrations. According to Figure 7b, the reverse solute flow (J_s_) also depended on the DS concentrations, where the J_s_ grew as the draw solution concentration increased. The highest J_s_ was 5.7 GMH at a DS concentration of 2.5 M. Additionally, the ratio of the solute flux to the water flux (J_s_/J_w_) increased with increasing draw solution concentrations (Figure 7c). Indeed, the salt rejection (SR%) reached a maximum of 98.8% at a 1 M concentration of the DS and was almost constant with further increases in the concentration of DS (Figure 7d). This may be attributed to the presence of TiO_2_, which increases the membrane porosity, reducing salt diffusion.

### 5.3. MMWFO Membrane (ST3) Application

#### 5.3.1. Effect of Different Feed Types on MMWFO Membrane Performance

**Figure 8:**
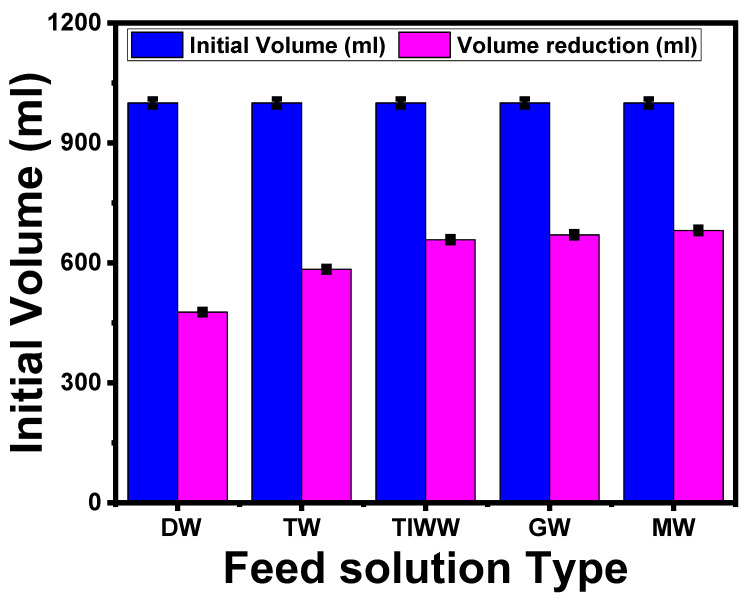
Effect of different feed solution types on the reduced volume. The standard deviation of measurements taken in triplicate is indicated by error bars.

Figure 8 depicts the results of using 1 M NaCl as the DS to show the influence of various feed sources, including distilled water (DW), tap water (TW), textile industrial wastewater (TIWW), gray water (GW), and municipal wastewater (MW), on the volume reduction for the optimized ST3 membrane. It was observed that the volume reduction of various feed types occurred in the following order: distilled water, tap water < industrial textile wastewater < gray water < and municipal wastewater. The driving force, which is another term for the osmotic pressure difference, is said to be responsible for these events. As a result, the driving power is highest when using pure water because there are no ions in the solution, whereas it decreases when using various types of wastewater.

Table 7 displays the analysis of various wastewater feed solutions (GW, TIMM, and MW) and a synthetic saline solution (1 M) after one hour of the FO process. For comparison, the major pollutants, including COD, TDS, TSS, and conductivity (Table 2), were present in higher amounts in the FS after 1 h (Table 7). A higher volume of penetrated water was transferred from the FS to the DS, which increased the concentration in the feed solution and demonstrated that there were no contaminants adhering to the membrane surface. The improvement of the FO membrane made with TNT particles, which increased the membrane porosity and hydrophilicity and reduced the contact angle, provides evidence for this phenomenon. Higher membrane performance was obtained at the ideal TNT content of 0.01% (ST3).

In general, according to Table 7, the volume reduction of wastewater depends on the used FS type, composition, and TDS concentration. In the following, ST3 exhibits the enhanced performance of saline water (1 M NaCl) for FO application on seawater, which gives high water flux with minimum reverse salt flux. The analysis of the DS indicated that there were no pollutants in the DS from different FSs used, and the DS concentration (1 M NaCl) after one hour was diluted to 0.51, 0.525, 0.59, 0.6, and 0.61 M, depending on the amount of permeated water.

#### 5.3.2. Effect of Time on ST3 Membrane Flux

**Figure 9:**
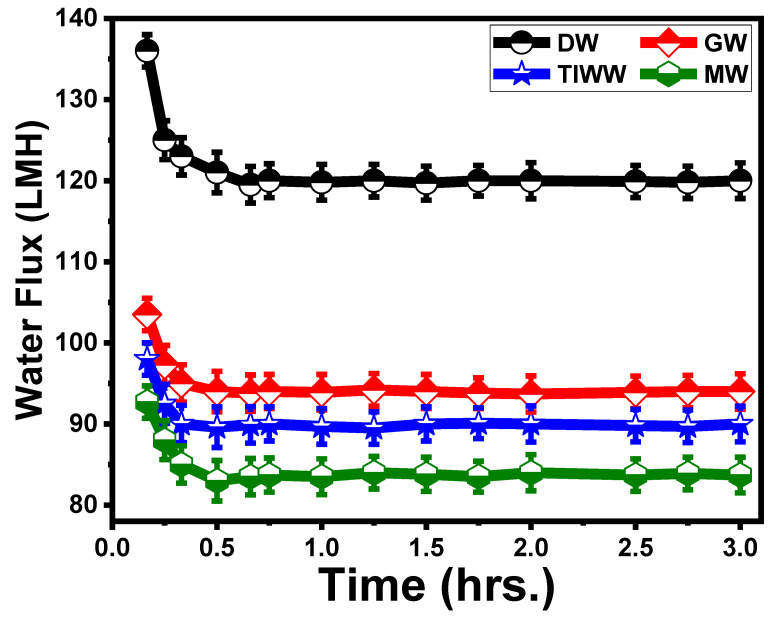
Effect of time on membrane fouling. The standard deviation of measurements taken in triplicate is indicated by error bars.

Figure 9 shows the effect of time on membrane fouling using different feed solutions. It is known that complex feed water can cause various types of fouling [79], including organic, inorganic, particulate, and biological fouling. In addition, the mechanisms of the water flux decline in the FO process are known to be more complicated due to ICP and RSF than those in the pressure-driven membrane process [29]. Because of this convoluted situation in the identification of the fouling mechanism, it is hard to clearly understand the fouling phenomenon. Thus, we focused on which membrane had greater fouling resistance and explored the possible reasons for this in this experiment. It is commonly known that lower initial flux and membrane roughness can reduce the extent of fouling in both pressure-driven and osmotically driven membranes [80].

In earlier investigations, the initial value of J_w_ for DW indicated the lowest fouling tendency, whereas the performance of the ST3 membrane with MW indicated the highest fouling tendency [81]. The initial J_w_ values for GW and TIWW were 103.5 and 98 LMH. The higher the initial flux is, the larger the hydrodynamic drag force toward the membrane and the higher concentration of polarization [82].

According to the critical flux concept, under the condition when the initial water flux is above the critical flux, foulants will start to deposit on the membrane surface, and water flux will start to decline. In the critical flux concept, the foulant–membrane interaction can significantly affect the critical flux [83]. In addition, the chemical characteristics (roughness, morphology, and pore size) of the membrane’s active layer can significantly influence the critical flux.

#### 5.3.3. Effect of Different NaCl Concentrations on the Volume Reduction of Textile Wastewater

**Figure 10:**
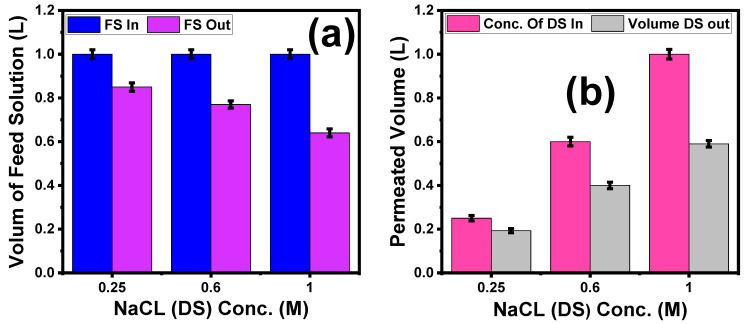
(**a**) Effect of NaCl (DS) concentrations on the volume of feed solution reduction and (**b**) effect of permeated volume of textile dye wastewater on dilution of different DS concentrations. The standard deviation of measurements taken in triplicate is indicated by error bars.

Figure 10a illustrates that with increasing DS concentrations from 0.25 M to 1 M, the volume reduction of textile wastewater increases from 0.85 L to 0.64 L. This result agrees with Phuntsho et al. [14,70], where the osmotic pressure between the DS and FS increased when increasing the DS solution concentration to the maximum at 1 M NaCl; i.e., the percentage of volume reduction increased from 15% at 0.25 M to 36% at 1 M NaCl. In addition, Figure 10b confirms this result, where the higher osmotic pressure difference between the FS and DS causes the volume of permeated water to increase from 0.193 L at 0.25 to 0.59 L at 1 M NaCl. For comparison, Table 8 shows the obtained results of the J_s_, J_w,_ and J_s_/J_w_ of our optimized membrane compared with various types of commercial, modified, and previously reported FO membranes [19,23,75,77,84,85,86,87,88,89,90,91,92]. Therefore, with a low cost and excellent efficiency, our optimized TiO_2_/Na_2_Ti_3_O_7_ nanocomposite/PES mixed-matrix woven membrane might be promising as a membrane for water treatment in both FO and PRO orientations.

## 6. Conclusions

Water scarcity is viewed as a severe issue, both globally and in Egypt specifically. To achieve sustainable development, an antifouling FO membrane must be developed (as an energy-efficient approach). One of the alternatives to address water shortages is water treatment. A TNT nanocomposite was synthesized using the hydrothermal process, and PES/TNT MMW FO membranes were produced using the phase inversion method with different TNT weight ratios (0.001 to 0.1 wt.%). The morphologies and structures of the TNT nanocomposite and PES/TNT MMW FO membranes were investigated using a variety of characterization techniques. The TNT nanocomposite with a forked nano-fur morphology consisted of monoclinic Na_2_Ti_3_O_7_ and anorthic TiO_2_ with good crystallinity, had a ($\overset{-}{2}11$) preferred growth orientation, and had an average crystallite size of ~60 nm. All produced PES/TNT membranes had irregular porous layer structures that extended from the middle to the bottom surface, with a dense top layer. The TNT doping ratio had an impact on the PES/TNT nanocomposite membranes’ pore size, and the highest porosity (74%) and lowest tortuosity (2.1) were observed for the ST3 membrane (0.01 wt.% TNT). The effect of the NaCl draw solution (DS) at different concentrations and the membrane orientation on the mixed-matrix membrane performance was investigated using distilled water (DW) as the feed solution (FS). The optimal FO performance was observed for the ST3 membrane, which had the highest water flux of 136 LMH, an RSF of 0.035 GMH, a salt rejection rate of 98%, and a volume reduction of 52%. Different water samples obtained from various sources were used as the feed solution and treated using the optimized ST3 membrane. The membrane demonstrated consistent water flow and showed antifouling behavior with the various sources of wastewater after 30 min of use with 1 M NaCl as the DS. Gray water, municipal wastewater, textile industrial wastewater, and tap water resulted in volume reductions of 41%, 34%, 33%, and 31.9%, respectively. Although a higher volume reduction of 41% was obtained when using NaCl as the DS and textile industry effluent as the FS, 1 M NaCl resulted in more permeated water than 0.25 M and 0.5 M. Finally, it can be claimed that these membranes represent a brand-new class of FO membranes that are suitable for use in wastewater treatment.

## Figures and Tables

**Table 1:** Composition of PES/TNT nanocomposite MMW FO membranes fabricated with different TNT nanocomposite wt.%.

Membrane ID	PES (wt.%)	NMP (wt.%)	TNT (wt.%)	PEG (wt.%)
Bare PES	22	77.5	0	0.5
ST1	22	77.496	0.004	0.5
ST2	22	77.492	0.008	0.5
ST 3	22	77.49	0.01	0.5
ST 4	22	77.46	0.04	0.5
ST 5	22	77.42	0.08	0.5
ST 6	22	77.4	0.1	0.5

**Table 2:** The average characteristics of different feeding water used.

Parameter	Textile Industrial Wastewater (TIWW)	Low-Strength Gray Water (GW)	Municipal Water (MW)
pH	7.5	7.7	7.3
COD g/L	1.194	0.28	0.39
TSS g/L	0.860	1	1.8
TDS g/L	0.56	0.585	0.6
Conductivity	1.084	1.018	1.052
PO_4_ g/L	0.09	0.009	0.14
Total Nitrogen g/L	-	-	0.04
Sulfide g/L	-	-	4.4

**Table 3:** The XRD structural parameters calculated for high peaks of anorthic TiO_2_ and monoclinic Na_2_Ti_3_O_7_.

Compound Name	Anorthic TiO_2_				Monoclinic Na_2_Ti_3_O_7_			
	( $0\overset{-}{1}3$)	(040)	( $\overset{-}{1}\overset{-}{3}3$)	(063)	( $\overset{-}{2}11$)	( $\overset{-}{3}02$)	( $\overset{-}{4}22$)	(520)
2θ (^o^)	18.33	27.57	29.20	45.63	31.91	34.03	66.37	75.47
d-spacing (Å)	4.84	3.23	3.05	1.99	2.80	2.63	1.41	1.26
I/I_o_ (%)	9.34	8.95	16.55	57.45	100	22.98	18.55	15.96
FWHM (β)	0.118	0.236	0.157	0.197	0.157	0.157	0.118	0.118
Microstrain × 10^−3^	3.19	4.20	2.64	2.04	2.40	2.24	0.79	0.67
D (nm)	71.14	36.16	54.47	45.72	54.81	55.11	83.91	88.80
TC	0.58	0.56	1.03	3.58	4.31	0.99	0.80	0.69
δ × 10^−3^ (dis/nm^2^)	0.20	0.76	0.34	0.45	0.33	0.33	0.14	0.13

**Table 4:** Surface roughness parameters of the synthesized PES/TNP MMW FO membranes fabricated with different TNP wt.% ratios.

Sample	Ra (nm)		Rq (nm)	
	Top	Bottom	Top	Bottom
Bare PES	16.5	14.6	18.3	16.7
ST2	16.7	15.8	18.4	18.5
ST3	17.9	19.4	19.8	20.8
ST6	17.5	14.9	17.9	15.4

**Table 5:** Effect of TNP wt.% on the water uptake %, porosity, contact angle, thickness, tortuosity, and S value of PES/TNP MMW FO membranes prepared using starting casting thickness of (A) 215 µm.

Samples	TNP Wt.%	Water Uptake (%)	Porosity (%)	Contact Angle (^°^)	Thickness (µm)	Tortuosity (τ)	S Value
Bare PES	0	49	45	78	167	5.3	19.6
ST1	0.004	64	54	73	166	3.9	11.98
ST2	0.008	70	60	68	153	3.2	8.16
ST3	0.01	77	74	63	152	2.1	4.3
ST4	0.04	52	52	58	153	4.2	12.1
ST 5	0.08	46	53	55	154	4.1	10.6
ST 6	0.1	30	31	51	154	9.2	45.7

**Table 6:** Comparison of calculated Jw, Js, Js/Jw, and SR% in both FO and PRO modes of the fabricated MMWFO membranes.

Parameter	FO (Al-FS) Mode				PRO (Al-DS) Mode			
	J_w_ (LMH)	J_s_ (GMH)	J_s_/J_w_ × 10^−3^	SR%	J_w_ (LMH)	J_s_ (GMH)	J_s_/J_w_ × 10^−3^	SR%
Bare PES	67	4.78	71.3	81	81	6.8	83.9	72
ST1	97	0.56	5.8	96	131	8.2	62.6	82.5
ST2	129	0.37	2.9	97.6	140	10.5	75	78
ST3	136	0.035	0.3	98.8	149	12.9	86.6	70
ST4	68.6	0.4	5.8	97	78	15.4	197	68.5
ST5	61.7	0.7	11.4	95	72	15.9	220	67.7
ST6	48.6	1.78	36.6	93.6	62	16	258	68

**Table 7:** Analysis of the different types of wastewater as the FS after 1 h using the FO process.

Parameter	Textile Industrial Wastewater (TIWW)	Low-Strength Gray Water (GW)	Municipal Water (MW)
pH	7.7	7.7	7.5
COD g/L	1.815	1.8	0.57
TSS g/L	1.307	1.3	0.26
TDS g/L	0.854	0.836	0.883
Conductivity	1.648	1.848	1.70

**Table 8:** Comparison of the performance of our optimized membrane with the performance of commercial and previously reported FO membranes in the literature.

Membrane	J_w_ (LMH)	J_s_ (GMH)	J_s_/J_w_ (g/L)	Test Conditions (FS/DS, Membrane Orientation)	Ref.
TiO_2_/Na_2_Ti_3_O_7_/PES	136	0.035	0.3 × 10^−3^	DI water/1 M NaCl, FO mode	This work
	149	12.9	86.6	DI water/1 M NaCl, PRO mode	
SiO_2_@MWNTs incorporated into polyvinylidene difluoride (PVDF)	22.10	4.10	0.19	DI water/1.0 M NaCl, FO mode	[84]
Polysulfone (PSF)/TiO_2_ nanocomposite	31.20	6.66	0.21	10 mM NaCl/ 0.5 M NaCl, PRO mode	[19]
NMP added to coagulation bath PES	62.70	10.30	0.16	DI water/2.0 M NaCl, PRO mode	[75]
p-TiO_2_ incorporated into polyvinylidene difluoride (PVDF)	18.70	4.50	0.24	DI water/1 M NaCl, FO mode	[85]
CaCO_3_ incorporated into PSF	25.40	57.00	2.24	DI water/2 M NaCl, FO mode	[86]
Zeolite incorporated into PSF	85	55.00	0.65	DI water/2 M NaCl, PRO mode	[23]
Polyamide thin-film composite membranes based on carboxylated polysulfone (TFC-cPSf flat sheet)	18	2.2	0.12	DI water/1 M MgCl_2_, FO mode	[87]
	27	5.5	0.09	DI water/1 M MgCl_2_, PRO mode	
Dual-layer polybenzimidazole-polyether sulfone (PBI-PES) nanofiltration (NF) hollow-fiber membranes	24.2	-	-	DI water/5 M MgCl_2_, FO mode	[88]
	33.8	-	-	DI water/5 M MgCl_2_, PRO mode	
Polyamide-imide (PAI) material as the porous substrate, followed by polyelectrolyte post-treatment using polyethyleneimine (PEI)	11.7	3.9	0.33	DI water/1.5 M MgCl_2_, FO mode	[89]
	17.3	16.6	0.96	DI water/1.5 M MgCl_2_, PRO mode	
(TFC) FO membrane with hydrophilic mineral (CaCO_3_)-coated polyether sulfone (PES)	52	16.8	0.32	DI water/2 M NaCl, FO mode	[77]
Polysulfone/polyacrylonitrile blend nanofibers as a porous substrate	38.3	10.1	0.27	DI water/1 M NaCl, FO mode	[90]
Thin-film nanocomposite (TFN) polyamide (commercial CTA-W)	14	5.6	0.4	DI water/1 M NaCl, FO mode	[91]
CTA-NW (commercial)	4.4	0.6	0.14	10 mM NaCl /0.5 M NaCl, FO mode	[92]
	8.19	2.8	0.34	10 mM NaCl /0.5 M NaCl, PRO mode	
CTA-HW (commercial)	9.03	5.3	0.59	10 mM NaCl /0.5 M NaCl, FO mode	
	15.4	9.4	0.61	10 mM NaCl /0.5 M NaCl, PRO mode	
CTA-W (commercial)	5	2.9	0.58	10 mM NaCl /0.5 M NaCl, FO mode	
	6.55	4.8	0.73	10 mM NaCl /0.5 M NaCl, PRO mode	

## Data Availability

The data presented in this study are available on request from the corresponding author.

## Supplementary Materials

The following supporting information can be downloaded at: https://www.mdpi.com/article/10.3390/membranes13070654/s1, Table S1: Characteristic of used woven fabric.
