# Supplementary material for: Fabrication of PES Modified by TiO2/Na2Ti3O7 Nanocomposite Mixed-Matrix Woven Membrane for Enhanced Performance of Forward Osmosis: Influence of Membrane Orientation and Feed Solutions
Source: Membranes (Basel). 2023 Jul 8;13(7):654. doi: 10.3390/membranes13070654 (PMC10383846; doi:10.3390/membranes13070654)
Supplement: Supplementary file 1 [file membranes-13-00654-s001.zip › membranes-2455487-supplementary.pdf]

Fabrication of PES modified by $TiO_2/Na_2Ti_3O_7$ nanocomposite mixed matrix woven membrane for enhancement performance of forward osmosis: Influence of membrane orientation and feed solutions

Ghadah M. Al-Senani[1], Mervat Nasr[2,3], Mohamed Zayed[2], Sahar S. Ali[4], Hind Alshaikh[5], Hanafy M. Abd El-Salam[3] and Mohamed Shaban[2, 6*]

[1] Department of Chemistry, College of Science, Princess Nourah bint Abdulrahman University, P.O. Box 84428, Riyadh 11671, Saudi Arabia; gmalsnany@pnu.edu.sa

[2] Nanophotonics and Applications (NPA) Lab, Physics Department, Faculty of Science, Beni-Suef University, Beni-Suef, 62514, Egypt; m.zayed88ph@yahoo.com (M.Z.); mervat.nasr@science.bsu.edu.eg (M.N.); mssfadel@aucegypt.edu (M.S.)

[3] Chemistry Department, Faculty of Science, Beni-Suef University, Beni-Suef 62514, Egypt

[4] Chemical Engineering and Pilot-Plant Department, National Research Center, Dokki, Cairo, 12622, Egypt; sahar_saad_ali@yahoo.com (S.S.A.)

[5]Chemistry Department, Science and Arts College, Rabigh Campus, King Abdulaziz University, P.O. Box 344, Jeddah 21911, Saudi Arabia; hfalshakh@kau.edu.sa

[6]Department of Physics, Faculty of Science, Islamic University of Madinah, P. O. Box 170, Madinah 42351, Saudi Arabia; mssfadel@aucegypt.edu (M.S.)

* Corresponding author: mssfadel@aucegypt.edu

Table S1. Characteristic of used woven fabric .

Fabric type	Fiber diameter (µm)	Thickness (µm)	Permeability ($L/m^2h.bar$)	Tensile strength (N/cm^2)	Elongation (%)	Contact angle (°)
**Cetin Woven**	11.6	115	1254	31.2	44.2	41
